# WGS based study of the population structure of *Salmonella enterica* serovar Infantis

**DOI:** 10.1186/s12864-019-6260-6

**Published:** 2019-11-15

**Authors:** Pernille Gymoese, Kristoffer Kiil, Mia Torpdahl, Mark T. Østerlund, Gitte Sørensen, John E. Olsen, Eva M. Nielsen, Eva Litrup

**Affiliations:** 1 Department of Bacteria, Parasites and Fungi, Statens Serum Institut, Copenhagen, Artillerivej 5 Denmark; 2 Department of Veterinary and Animal Sciences, University of Copenhagen, Stigbøjlen 4, Frederiksberg C, Denmark

**Keywords:** Salmonella, Infantis, Population structure, Bacteriophages, Evolution, Diversity, Prophage, Whole genome sequencing

## Abstract

**Background:**

*Salmonella* Infantis (*S*. Infantis) is one of the most frequent *Salmonella* serovars isolated from human cases of salmonellosis and the most detected serovar from animal and food sources in Europe. The serovar is commonly associated with poultry and there is increasing concern over multidrug resistant clones spreading worldwide, as the dominating clones are characterized by presence of large plasmids carrying multiple resistance genes. Increasing the knowledge of the *S*. Infantis population and evolution is important for understanding and preventing further spread.

In this study, we analysed a collection of strains representing different decades, sources and geographic locations. We analysed the population structure and the accessory genome, in particular we identified prophages with a view to understand the role of prophages in relation to the evolution of this serovar.

**Results:**

We sequenced a global collection of 100 *S*. Infantis strains. A core-genome SNP analysis separated five strains in e-Burst Group (eBG) 297 with a long branch. The remaining strains, all in eBG31, were divided into three lineages that were estimated to have separated approximately 150 years ago. One lineage contained the vast majority of strains. In five of six clusters, no obvious correlation with source or geographical locations was seen. However, one cluster contained mostly strains from human and avian sources, indicating a clone with preference for these sources. The majority of strains within this cluster harboured a pESI-like plasmid with multiple resistance genes. Another lineage contained three genetic clusters with more rarely isolated strains of mainly animal origin, possibly less sampled or less infectious clones.

Conserved prophages were identified in all strains, likely representing bacteriophages which integrated into the chromosome of a common ancestor to *S*. Infantis. We also saw that some prophages were specific to clusters and were probably introduced when the clusters were formed.

**Conclusions:**

This study analysed a global *S*. Infantis population and described its genetic structure. We hypothesize that the population has evolved in three separate lineages, with one more successfully emerging lineage. We furthermore detected conserved prophages present in the entire population and cluster specific prophages, which probably shaped the population structure.

## Background

*Salmonella enterica* is a frequently reported zoonotic bacteria causing many cases of gastroenteritis worldwide [1]. The species consists of six subspecies and these subspecies can be divided into more than 2500 serovars [2]. *Salmonella enterica subsp. enterica* serovar Infantis (*S*. Infantis) is one of the top ten serovars causing human salmonellosis in both Europe and North America [3, 4]. In Europe, *S*. Infantis is the most frequently reported *Salmonella* serovar from animal and food sources, with the majority of strains found in the poultry production chain [3]. *S*. Infantis is considered a target organism for regulation in breeding flocks in the EU and is estimated to account for 38.6% of all isolated serovars from *Gallus gallus* [3]. Also, in the US, *S*. Infantis is in the top ten of the most prevalent serovars associated with poultry [5]. However, this serovar is also isolated from various other sources, with swine being one of the more frequent [6, 7].

*S.* Infantis populations have been characterized in several countries [8–14]. However, most of these studies have limited their investigations to national isolates, often only from certain reservoirs, and they have limited the time periods or selected strains based on their resistance profiles. Thus, there is clearly a lack of knowledge about the overall population structure of this important *Salmonella* serovar.

In recent years, antimicrobial resistance has increased in *S.* Infantis strains circulating the poultry industry, where *S*. Infantis accounts for a large proportion of the overall number of multidrug resistant *Salmonella* strains [15]. Large conjugative plasmids, carrying multiple resistance genes, have been associated with this development [11, 12, 16–18], but it remains to be shown how common these are in the population of *S.* Infantis.

Prophages are bacteriophages that have become resident parasites in the bacterial host genome. The host genome can contain several prophages depending on host genus and the resident prophages, and the prophages can be active or only remnants of active prophages. Active prophages can excise from the bacterial genome and form new bacteriophages that can work as a weapon and kill other bacteria [19]. Additionally the prophage can bring resistance genes, virulence genes and other cargo genes, which can potentially be an advantage to the host [20–22]. All of these interactions between bacteriophage and bacteria can drive the evolution of certain clones by providing a competitive advantage over established clones. So far, no studies have specifically characterized the prophage reservoir in *S.* Infantis.

The aim of the current study was to provide an overview of the global population structure of *S.* Infantis and examine the presence of prophages in relation to the evolution of this bacterial pathogen, by using prophages as markers for strain divergence in the population. For this purpose, we sequenced a diverse strain collection consisting of 100 strains and we examined the *S.* Infantis population structure. Furthermore, we analysed the prophage content and determined the presence of large, multi-drug resistance plasmids.

## Results

### Population structure

We analysed the phylogenetic relationship between 105 *S.* Infantis strains based on 28,860 identified core-genome SNPs. Our results showed that five strains were clearly separated with a long branch (Additional file 2: Figure S1). The five separated strains were isolated from Africa, Asia and Europe and were sequence type (ST) 493, 603 and 1823, having 0–2 shared alleles with the most commonly isolated ST32. Sequence type 603 and 1823 belong to e-Burst Group (eBG) 297, whereas ST493 is not defined in an eBG. There were no shared alleles with eBG297 and ST493. Throughout the rest of our study, we chose to focus on the remaining 100 strains consisting of 97 ST32 strains and 3 strains that were single locus variants of ST32 (ST1032, ST1824 and ST1825), all belonging to eBG31.

**Fig. 1:**
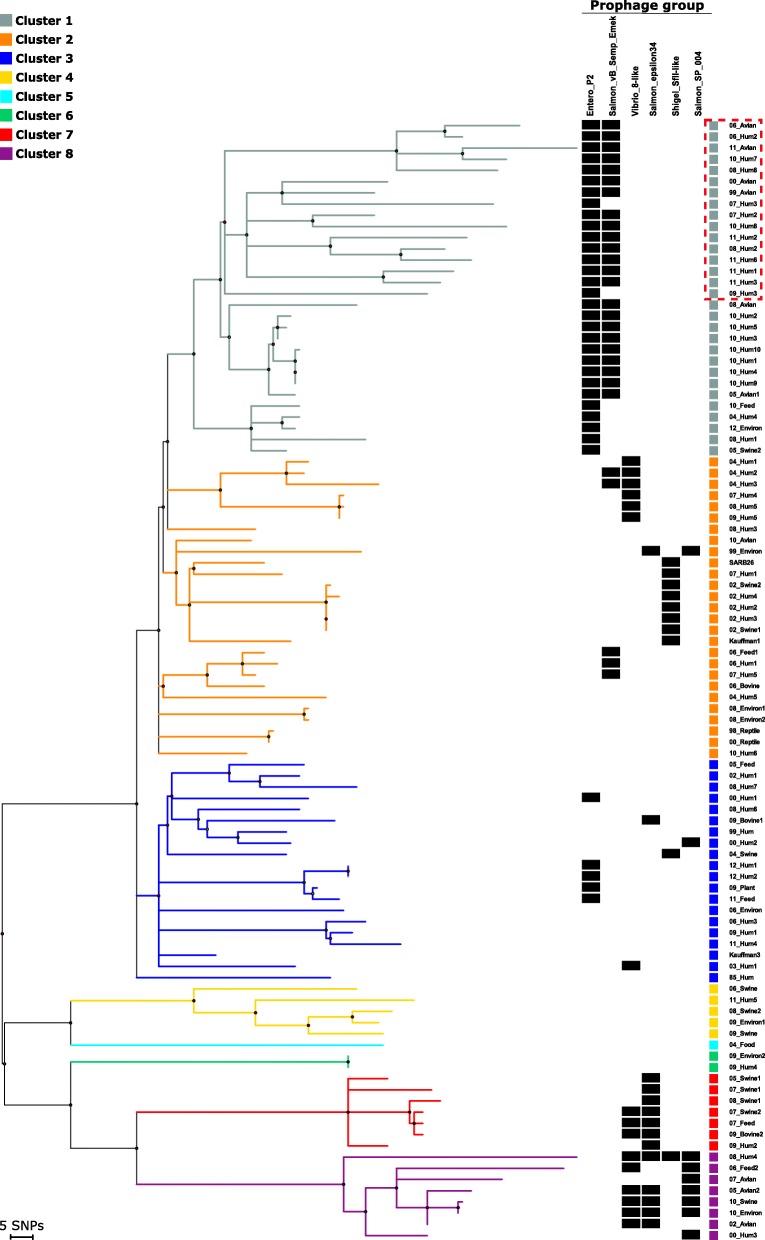
Phylogenetic tree based on core-genome SNPs of 100 *S.* Infantis strains. Maximum parsimony tree based on 2311 core-genome SNPs in 100 *Salmonella* Infantis strains. Branches are coloured according to clusters identified from STRUCTURE analysis and branch length correlates with SNP distances. Each strain is designated by year of collection and source type and coloured according to cluster. The presence of prophages are marked with a black box. The four prophages that are present in all genomes are excluded from the figure, as are the prophages detected in less than ten genomes. Stains with pESI-like plasmid are marked with a red dotted box

The core-genome SNP analysis from the remaining strains resulted in 2311 SNPs. Based on this SNP matrix we calculated a phylogenetic tree and defined eight genetic clusters (cluster 1–8) within the collection, where cluster 1 seems to be a subcluster of cluster 2 (Fig. 1). The clustering was confirmed by a STRUCTURE analysis (Additional file 2: Figure S2), however low or questionable cluster membership probability values (Q-values) were observed for nine strains (Additional file 1: Table S2). Roughly, the population could be divided into three lineages, a main lineage with clusters 1–3 having the most strains, an intermediary lineage with clusters 4 and 5 and a distant lineage of clusters 6, 7 and 8 (Fig. 1).

BEAST analysis on the population showed that clusters 1–5 and clusters 6–8 have evolved as separate lineages from a common ancestor around 150 years ago (Additional file 2: Figure S3). The distant lineage had an early branch (cluster 6) and was shown to further separate into clusters 7 and 8 around 100 years ago. Furthermore, the analysis showed branching of the main lineage into clusters 1–3 approximately 75 years ago. Clusters 4–5 (intermediary isolates) seemed to have split from the main lineage more than 100 years ago.

Overall, the clustering in this study did not show a clear correlation to the geographical origin of the strain. We detected strains isolated in Denmark in every cluster in the tree (except cluster 5, which was a singleton). A SNP analysis was made including all genomes from a Japanese study and it showed a good correlation between the clustering defined in our study and the published study from Japan [23] (Additional file 2: Figure S4). We included one representative genome from each Japanese cluster into our further analysis.

In clusters 2–6, we did not observe any clear correlation between clustering of strains and sources, however we did observe trends in cluster 1 and cluster 7 and 8. Cluster 1 had a majority of strains isolated from humans or related to poultry. Only a single swine related strain was detected in this large cluster consisting of almost one third of the strains in this study. To test whether this cluster represented a clone with a preference for infecting avian sources as opposed to porcine sources, an additional SNP analysis was performed on our collection with the inclusion of published genomes of 50 porcine and 50 avian strains. The analysis placed an additional 14 poultry related strains in our cluster 1 and only a single strain isolated from swine (Additional file 2: Figure S5).

Cluster 7 and 8 were clearly separated from the remaining clusters with a longer branch (Fig. 1). The two clusters consisted of 15 strains, three of which were isolated from human (1 from an asymptomatic carrier) and 12 strains were isolated from swine, poultry, feed and the environment. We speculated whether these clusters could be a group of less virulent strains due to the overrepresentation of strains isolated from animals and the environment. Hence, we included the genomes from our study in an analysis with 7852 public *S*. Infantis genomes (Additional file 2: Figure S6). The analysis resulted in merely 179 additional genomes of both human (93 genomes), animal (47) and unknown (39 genomes) sources clustering with strains from our distant outlier lineage. The remainder of the public genomes clustered together with the rest of our *S.* Infantis population.

A total of 25 strains in our study were multidrug resistant (MDR) with resistance to three or more antimicrobial classes as defined by Magiorakos et al. 2012 [24]. A sub cluster of 16 strains within cluster 1 contained only strains that were resistant to multiple antimicrobials, which was in contrast to the other *S.* Infantis strains in our study, where the majority were susceptible. In this sub cluster, all 16 strains were resistant to streptomycin, sulfamethoxazole, ciprofloxacin and nalidixic acid and some strains were furthermore resistant to tetracycline, trimethoprim and neomycin (Additional file 1: Table S1). A mapping against the megaplasmid pESI described by Aviv et al. [18] revealed that all 16 strains harboured a pESI-like plasmid of approx. 280–283 kb, with 15 strains having 95.9–99.8% sequence identity with the pESI plasmid and one strain having 81.3% sequence identity. Within the plasmid related regions, resistance genes for streptomycin (*aadA1*), sulfamethoxazole (*sul1*) and tetracycline (*tetA*) were detected. A region of approximately 3 kb harbouring the gene for trimethoprim resistance (*dfrA14*) was identified in nine out of the 16 strains. The plasmids did not contain any *bla-*_CTX-M-1_ or *bla*-_CTX-M-65_ genes as reported in the ESBL clones with pESI-like plasmids detected in Italy, USA and Switzerland [11, 12, 16].

### Prophages

**Fig. 2:**
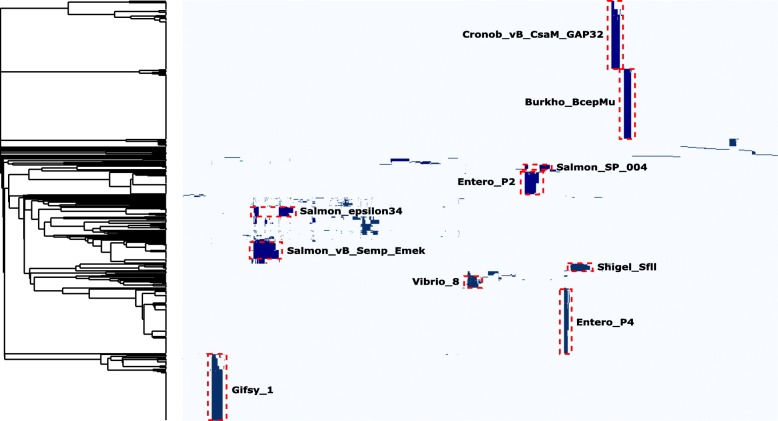
Gene-by-gene comparison of prophages identified in 100 strains of *S.* Infantis. Heatmap of gene-by-gene comparison of prophages identified in 100 strains of *Salmonella* Infantis. The phylogenetic tree is based on single linkage clustering of jaccard dissimilarities of the prophages based on gene presence/absence. Presence of genes is marked with dark blue and the ten defined prophage groups are marked in boxes with red dotted lines

In the entire collection of 105 *S.* Infantis genomes we detected 634 prophages, ranging from 4 to 8 prophages per genome. The prophages were divided into groups based on a gene-by-gene comparison illustrated by the heatmap shown in Fig. 2. The majority of prophages (84%) were detected in 10 or more genomes and divided into the 10 prophage groups listed in Table 1. The remaining prophages (16%) were detected in fewer strains and showed limited similarity to other prophages in the population. Four prophages were present in all strains, including the five strains with the distantly related ST’s. Out of these four conserved prophages three were incomplete. For the remaining six prophage groups, we saw that some were primarily detected in one specific cluster, whereas other prophage groups were detected more sporadically in up to four different clusters (Fig. 1). In general, we saw nearly 100% sequence similarity for prophages present in genomes within a specific cluster. In clusters 7 and 8, an identical Salmon epsilon34-like prophage were found in 12 out of 16 genomes. Additionally, in cluster 8 we also found a Salmon SP-004-like prophage present in seven out of eight genomes. All strains in cluster 1 contained an Entero P2-like prophage and in 23 out of 30 genomes we found a prophage identified as Salmon vB Semp Emek-like (Fig. 1). Both of these prophages were not exclusively identified in cluster 1, however both of the prophages were detected in only five other genomes in the entire collection.

**Table 1:** Prophages identified in a collection of 105 *Salmonella* Infantis strains

Prophage group	No. of isolates	% of total no of prophages	Close related prophage^a^
Burkho_BcepMu	105	17	*Burkholderia* phage BcepMu
Cronob_vB_CsaM_GAP32	105	17	*Cronobacter sakazakii* phage GAP32
Entero_P4	105	17	Enterobacteria phage P4
Gifsy_1	105	17	Phage Gifsy-1
Entero_P2	35	6	Enterobacteria phage P2
Salmon_vB_Semp_Emek	28	4	*Salmonella* phage vB_SemP_Emek
Vibrio_8	16	3	*Vibrio* phage VPUSM 8
Salmon_epsilon34	14	2	*Salmonella* phage epsilon34
Shigel_SfII	11	2	*Shigella* phage SfII
Salmon_SP_004	10	2	*Salmonella* phage FSL SP-004
Ungrouped prophages	100	16	–

^a^ Based on output from PHAST analysis

## Discussion

*Salmonella* Infantis is reported as an increasingly isolated serovar in many countries worldwide [25, 26], with special focus on new circulating clones having a pESI-like plasmid with multiple antimicrobial resistance genes [11, 12, 16–18]. To better understand this serovar and how it evolves, this study examined a global population of *S.* Infantis strains isolated from different sources over several decades. Based on the analyses, we concluded that *S.* Infantis is a polyphyletic serovar. The polyphyletic structure is often seen in the *S. enterica* subsp. *enterica* population where most serovars have developed in discrete starburst-like clusters [27]. The fact that we observe strains with the *S*. Infantis antigenic formula not located in the eBG31 could be due to the moderately frequent recombination that has been described in *Salmonella* [28–30].

The core-genome SNP analysis of the selected 100 strains from eBG31 resulted in eight clusters. The grouping was solid and supported by both core-genome SNP, STRUCTURE and BEAST analysis. Other studies reported *S*. Infantis clusters ranging from two closely related genotypes [8, 31] to three, four or five clusters in the investigated population [13, 14, 32]. These previous studies were based on PFGE and the lower number of clusters corresponds well to the method being less discriminatory, but could also be influenced by the limited sampling done in these studies. Another study based on WGS of a collection of *S*. Infantis strains, that exclusively were isolated in Japan, detected five clusters in their population based on SNP analysis [23]. Based on the inclusion of the Japanese strains in our study, we observed that the five clusters detected in the Japanese study correlated to four of our clusters.

The evolutionary BEAST analysis showed that the serovar evolved into two lineages from a common ancestor around 150 years ago, and that the main lineage with the majority of strains expanded around 75 years ago. While this expansion probably cannot be explained by any single event, the timing correlated well with the increased industrialization and specialization of livestock production in the mid-1900. However other changes in climate and human migration could also have influenced the expansion of *S*. Infantis.

Our study identified two distant clusters mostly of non-human origin. Initially, the lack of human strains made us speculate whether this lineage was more adapted to animals and less virulent to humans. After comparison to the publicly available *S.* Infantis, only relatively few genomes were included in clusters 7 and 8, and amongst these we observed a higher proportion of human genomes than seen in this study. Therefore, a more likely explanation was that these distant clusters represent strains that are not typically sampled and therefore not present in our collection and the public genome collections. The strains in these distant clusters generally represents a smaller threat to the food production animals and environment and therefore subsequently to humans.

No obvious correlation of clustering with geography was seen in this study and the known diversity of *S*. Infantis seems to be represented worldwide. Some of this diversity may reflect the global penetration of just a few poultry breeding companies. In accordance with this conclusion, strains isolated in Denmark were present in nearly all clusters in the tree, just as strains isolated in Japan from the study of Yokoyama et al. [23] were present in four clusters in the tree. A potential source of error in our strain collection is the fact that we attributed a country of travel as the origin of the infection and thereby origin of the strain. In spite of the information regarding country of travel, we cannot be certain these infections were acquired abroad.

Cluster 1 consisted of strains of mainly human and avian origin and only one strain related to swine, indicating a clone likely favouring the avian reservoir. The relation to avian and human sources was supported by an additional SNP analysis with inclusion of genomes from porcine and avian sources. We speculate that this cluster has evolved as a type which is more established in the avian reservoir and thus infecting humans. Further analysis, including detailed analysis of the accessory genome and biological studies with colonization of animals, are needed to determine further host relatedness.

Prophages have long been known to contribute traits that drive the evolution of bacteria [33]. In order to analyse the presence of bacteriophages in the *S.* Infantis population we determined the content of prophages in our strain collection. Four prophages were detected in all strains in our population, suggesting that these were integrated into the genomes of *S*. Infantis in an ancestor common with other *Salmonella* serovars. Similar prophage sequences (from BLAST search against the NCBI database) were seen in several other *Salmonella* serovars e.g. Typhimurium, Newport, Kentucky, Anatum, Tennessee, Senftenberg, Enteritidis and Thompson. The prophages are present in a broad range of serovars from *S. enterica* subsp. *enterica*, leading us to conclude that these prophages were introduced early in the formation of this subspecies even before the individual serovars originated.

Three of these prophages were incomplete, corresponding well with the theory that prophages are rapidly degraded after integration into the host genome and subsequently stabilized in the genome in a smaller and non-complete version [34]. The remainder of the detected prophages were present in a range of 33% of genomes down to just one genome. The larger prophage groups were primarily located in specific clusters in the phylogenetic tree, leading us to conclude that these prophages were integrated into the genomes in events occurring at the same time as the branching of the tree. This suggests that prophages have been important in shaping the population structure of *S*. Infantis, an observation that supports previous observations based on studies of prophages in *Salmonella* serovars Typhi, Heidelberg and Enteritidis [35–37].

One prophage (Entero P2-like) was primarily detected in cluster 1, which harboured strains closely related to the dominant poultry clones detected throughout Europe. The Entero P2-like phage identified in this study showed high sequence similarity (98% identity and 79% query cover) with prophage Escher pro483, a prophage isolated from an avian pathogenic *Escherichia coli* (KR073661.1). Sequence alignment of the two prophages, showed variance in some prophage related proteins and in hypothetical proteins. It has previously been shown that P2 prophages can carry genes beneficial for the host, such as the *sopE* gene important for the success of emerging clones [35, 38, 39], and this could be the case in this poultry cluster.

The possible beneficial role of the remaining cluster specific prophages is yet to be examined and the functions of the detected hypothetical proteins are so far unknown.

Several studies have reported emergence of *S.* Infantis clones having plasmids with a pESI-like backbone carrying multiple resistance genes, most of these were associated with poultry sources [10–12, 16, 18, 40]. In 2007, an emerging broiler associated MDR clone of *S*. Infantis was reported in Hungary, harbouring a large plasmid with resistance genes for streptomycin [41]. The pESI plasmid was later identified and characterized in Israel by Aviv et al. [18] in isolates from 2008, and subsequently clones having a pESI-like plasmid have been reported in several countries, all with resistance against multiple drugs, including some with resistance against β-lactams [11, 12, 16]. Our cluster 1 harboured strains with a pESI-like plasmid carrying multiple resistance genes and with two different resistance profiles. Strains were isolated from 1999 to 2011 and from various geographical locations. The core-genome SNP analysis showed that these strains clustered together and furthermore, the evolutionary analysis indicated a separation of this cluster around 60 years ago. We suggest that the clones have been present in the poultry industry for a longer time and that the use of antimicrobials in the industry has selected for this clone and the uptake of the resistance plasmids. In Denmark, aminoglycosides, sulfonamides, trimetroprim and tetracyclines are used in the poultry industry, where tetracycline is the most commonly used antimicrobial in broiler flocks [42]. These antimicrobials are also administrated to food production animals in other countries [43, 44]. The usage of these antimicrobials could positively select for the MDR clones and cause the rapid spread. This has also lead to concern for further spread in EU, where *S*. Infantis accounts for a large proportion of the overall number of MDR *Salmonella* [15].

In conclusion, our results suggested that even though clusters are readily identified based on SNPs in the core-genome, most of the intra serovar variation detected in *S.* Infantis are caused by prophage elements and plasmids.

## Conclusions

The *S.* Infantis serovar is polyphyletic and consists of several lineages harboring clones more or less widespread in the farm to fork chain. One lineage seems to consist of less sampled strains and represented by very few genomes when accessing the publicly available *S*. Infantis population. Another lineage contains a cluster that arose approximately 75 years ago which consists of a widespread clone that seems to have great success in infecting poultry and subsequently humans. We speculate that prophages play a major role in the evolution of this *Salmonella* serovar, and show that several prophages are specific for some clusters and others are inherent to most serovars in subspecies *enterica*.

## Methods

### Strain selection

The strains analysed in this study are listed in Additional file 1: Table S1. We selected 100 strains of *S*. Infantis of which 83 strains were from the Danish strain collections at Statens Serum Institute (SSI) and the National Food Institute (DTU) and 17 strains were from University of Warwick, UK. The collection at SSI included 56 human strains, of which 49 were from Denmark whereof 25 strains were isolated from returning travellers. Five whole genome sequences (Accession no. DRR022720, DRR022721, DRR022737, DRR022757 and DRR022768) from the study of Yokoyama et al. [23] were also included in the main collection analysed in this study . The strains were isolated from humans (*n* = 56), swine (*n* = 12), avian (*n* = 8), environment (*n* = 8), feed (*n* = 6), bovine (*n* = 3), reptile (*n* = 2), plant (*n* = 1), unknown food (*n* = 1) and unknown source (*n* = 3) (Additional file 1: Table S1).

The strains were selected to represent the known diversity by MLST and also to represent strains of both human and veterinary origin from Denmark, as well as travel related cases representing five continents. The collection included strains from year 1943 to 2012. Additional whole genome sequences for supportive analyses during the study were searched for and sorted in Enterobase [45] and sequence reads were downloaded from the sequence reads archive SRA [46]. Downloaded additional genome sequences included all sequences from the study of Yokoyama et al. [23], 50 genomes collected from swine and 50 genomes collected from avian sources (selected by ST32, sources and geography). Further 7852 *S*. Infantis genomes with HC200 = 36 (based on cgMLST V2 + HierCC) were selected in Enterobase for a large supportive analysis of the public available genomes.

### Antimicrobial susceptibility

Susceptibility to a standard panel of antimicrobial agents [47] was determined by microbroth dilution and interpreted using EUCAST ECOFFs [48] except for ciprofloxacin (> 0.125 μg/mL was used as breakpoint). The antibiotic resistance genes were determined from de novo assembled genomes using ResFinder 3.2 [49].

### Whole genome sequencing

Whole genome sequencing was performed on 74 strains at the University of Toronto in Canada. DNA was extracted and prepared by using the robotic setup described previously [50]. The genomes were sequenced using an Illumina GAIIx on 250 bp paired-end libraries in 8-fold multiplexes. The remaining 26 strains were sequenced at SSI in Denmark. DNA was extracted and prepared using Promega Wizard Genomic DNA Purification kit (Promega, Madison, USA) and Nextera XT v2 DNA Library Preparation kit (Illumina, San Diego, USA) according to the manufacturer protocol. Whole genome sequencing was performed using an Illumina MiSeq with 250 bp paired-end technology. All genomes were de novo assembled using CLC Genomic Workbench (Qiagen, USA).

### MLST

Sequence types were extracted from the de novo assembled genomes using MLST software [51] and named according to the seven gene MLST scheme for *Salmonella enterica* [52].

### Cluster analysis

Core-genome SNPs were detected using the NASP-pipeline [53]. SNPs were aligned and called against the complete reference genome of *S.* Infantis CVM44454 (CP016412.1) using BWA-MEM and GATK [54, 55]. Duplicate regions were masked and SNPs were filtered with a minimum coverage of 10 and a minimum proportion of 0.9. Clean core-genome SNPs used for cluster analysis in this study were defined as the SNPs passing the given filters and present in all genomes. Recombination events were removed using the tool CleanRecomb [56].

Multiple alignment of SNPs and calculation of maximum parsimony trees with bootstrap resampling of 200 were calculated in BioNumerics 7.6 (Applied Maths, Sint-Martems-Latem, Belgium). Rapid neighbour joining tree (RapidNJ) based on cgMLST was calculated using Grapetree in Enterobase [45, 57].

The strains were divided into clusters based on a STRUCTURE 2.3.4 analysis [58, 59] and on the phylogenetic tree. The analysis in STRUCTURE was run on the core-genome SNP matrix with the admixture ancestry model and the correlated allele frequencies model with a length of the burning period of 25,000 and a number of Markov Chain Monte Carlo repetitions (MCMC) of 25,000. Each run with a selected number of clusters (K) was repeated 20 times and the best number of clusters were evaluated using the online tool STRUCTURE HARVESTER 0.6.94 [60]. Out of the 20 repetitions for the best K, the run with the highest log likelihood value was used for estimating the affiliation of the isolates to the determined clusters based on the probability values (Q) and on the phylogenetic structure of the population. Cluster membership was considered to be questionable with Q-values below 0.8. The collection were separated in two STRUCTURE runs (the distant branch containing 15 strains and the remaining 85 strains clustering in the phylogenetic tree) to ensure a more precise analysis.

The evolution of the population was examined using BEAST 1.8 analysis on the SNP matrix [61]. An additional strain LN649235.1 was included in the analysis to give a more precise timeframe on the collection and on the distant lineage (the strain was located in cluster 8 and was collected from a healthy chicken in 1973). Two strains were excluded from this analysis due to no collection date (SARB26 and Kaufmann1). Analysis was run with 12 different model compositions of substitution models (GTR - general time reversible and HKY – Hasegawa-Kishino-Yano), clocks (S - strict and R - relaxed) and population structure (BS - baysian skyline, EP - exponential growth and CP - constant population size). The MCMC were set to 400.000.000 repetitions and with log every 40.000. The BEAST output was evaluated in Tracer 1.5.0 [62] and the best-fitted model was chosen based on the calculated Bayes Factor (BF – ratio of the marginal likelihood from 1000 recalculations) between models. Calculation of a mean phylogenetic tree from the best-fitted model was done in TreeAnnotator 1.8.4 (part of BEAST package) with a burning of 1000 trees, maximum clade credibility and medium node height.

### Identification of putative prophages

Prophage-like regions were identified using PHAST [63] on the de novo assembled contigs individually. Sequence contigs having less than 5000 bp were not analysed. The putative prophages were assigned a completeness score which was calculated based on the region size and numbers of phage-like genes. The prophages identified were named accordingly to the most probable known prophage found by PHAST. The identified prophage sequences were extracted from the assembled genomes using an in-house Python script. The extracted prophage sequences were annotated using Prodigal 2.6.3 [64] and Prokka 1.13 [65]. Genes were clustered into gene families using Roary 3.12.0 [66]. The prophage sequences were compared based on the Jaccard dissimilarity of the presence of gene families from the roary analysis and clustered using single linkage clustering. A heatmap of the absence/presence data was created using a modified version of Roary_plots.py [67]. The prophage sequences were furthermore aligned in BioNumerics. Prophages were finally assigned to prophage groups based on the PHAST output, the sequence similarity from the Bionumerics analysis and the gene-by-gene comparison. Groups were established if 10 or more genomes contained similar prophages.

### Identification of plasmids

Plasmids were identified from the de novo assembled genomes using PlasmidFinder 1.3 [68]. The mega plasmid pESI (project NZ_ASRF01000100) was downloaded from NCBI and reads for 16 strains were mapped against the plasmid using BWA-MEM, SAMtools, and GATK [54, 55, 69]. Vcf files were parsed to inspect mapping quality and positions with a depth 10 or greater were kept.

## Supplementary information

**Additional file 1: Table S1.** Strain information on 105 strains of *Salmonella* Infantis. **Table S2**. Q-values from STRUCTURE analysis of 100 strains of *Salmonella* Infantis.

**Additional file 2: Figure S1.** Maximum parsimony tree of 105 strains of *Salmonella* Infantis based on 28,860 core-genome SNPs with *Salmonella* Infantis CVM44454 as the reference genome. Branches are labelled with the number of SNP differences. Strains belonging to E-Burst Group (eBG) 31 and 297 are marked in red circles. **Figure S2.** Q-plots based on probability values (Q) from STRUCTURE analysis of 2311 core-genome SNPs identified in 100 *Salmonella* Infantis strains with *Salmonella* Infantis CVM44454 as the reference genome. Genetic clusters are marked with curly brackets and cluster number. A: STRUCTURE analysis on main lineage with 85 strains B: STRUCTURE analysis on distant lineage with 15 strains. **Figure S3.** Mean evolutionary tree calculated from BEAST analysis with the best-fitted substitution model (GTR-BS-R) on 2311 core-genome SNPs. Branches are coloured according to clusters and branch length correlates with time in years. **Figure S4.** Maximum parsimony tree of 167 strains of *Salmonella* Infantis based on 3454 core-genome SNPs with *Salmonella* Infantis CVM44454 as the reference genome. The collection of strains includes the 100 strains examined in this study, all genomes from Yokoyama et al. [23] (labelled Japan-clusters) and additional 6 genomes from SRA belonging to the distant lineage (cluster 7 and 8). Nodes are coloured according to clusters. **Figure S5.** Maximum parsimony tree of 200 genomes of *Salmonella* Infantis based on 4079 core-genome SNPs with *Salmonella* Infantis CVM44454 as the reference genome. The collection includes the 100 strains examined in this study and additional 50 genomes isolated from avian sources and 50 genomes from swine (downloaded from SRA). Nodes are coloured according to clusters and source. **Figure S6.** Core genome-derived phylogeny of 105 strains of *Salmonella* Infantis including additional 7852 public available *S*. Infantis genomes from Enterobase. RapidNJ tree based on cgMLST. Nodes are coloured according to clusters defined in this study.

## Data Availability

The whole genome sequences are available at the European Nucleotide Archive (ENA) under PRJEB30335. Five whole genome sequences (Accession no. DRR022720, DRR022721, DRR022737, DRR022757 and DRR022768) from the study of Yokoyama et al. [23] were also included in the main collection analysed in this study. The *S.* Infantis workspace in Enterobase used to produce the HC200 = 36 tree (based on cgMLST V2 + HierCC V1) in Additional file 2: Figure S6 is publicly available at http://enterobase.warwick.ac.uk/species/senterica/search_strains?query=workspace:33913.

## Abbreviations

BF: Bayes Factor
BS: Baysian Skyline
CP: Constant Population
eBG: e Burst Group
EP: Exponential growth
GTR: General Time Reversible
HKY: Hasegawa Kishino Yano
K: Number of clusters
MCMC: Markov Chain Monte Carlo
MDR: Multi Drug Resistant
MLST: Multi Locus Sequence Typing
Q: Probability values
R: Relaxed
S: Strict
SNP: Single Nucleotide Polymorphism
ST: Sequence Type
WGS: Whole Genome Sequencing
