# Supplementary material for: WGS based study of the population structure of Salmonella enterica serovar Infantis
Source: BMC Genomics. 2019 Nov 15;20:870. doi: 10.1186/s12864-019-6260-6 (PMC6858691; doi:10.1186/s12864-019-6260-6)
Supplement: Supplementary file 1 — Additional file 1: Table S1. Strain information on 105 strains of Salmonella Infantis. Table S2. Q-values from STRUCTURE analysis of 100 strains of Salmonella Infantis. [file 12864_2019_6260_MOESM1_ESM.pdf]

Table S1

Strain ID	Year	Host	Host details	Country attributed from travel	Country of isolation	MLST - 7 gene	Phenotypic resistance profile	ResFinder v. 3.2.	PlasmidFinder	Cluster
00_Avian	2000	Avian	Chicken meat	Japan	Japan	32	SSuTTmNe	aph(3')-Ia,aadA1,sul1,tet(A),dfrA14	IncFIB(pENTAS01)-pSEI-like	cluster 1
00_Hum1	2000	Human		Unknown	Unknown	32	Sensitive			cluster 3
00_Hum2	2000	Human	Asymptomatic carrier	Japan	Japan	32	Sensitive			cluster 3
00_Hum3	2000	Human	Asymptomatic carrier	Japan	Japan	32	Sensitive			cluster 8
00_Reptile	2000	Reptile	Snake	Germany	Germany	32	Sensitive			cluster 2
02_Avian	2002	Avian	Imported duck	Denmark*	Denmark*	32	Sensitive			cluster 8
02_Hum1	2002	Human	Faeces	Denmark	Denmark	32	Sensitive			cluster 3
02_Hum2	2002	Human	Blood	Denmark	Denmark	32	Sensitive			cluster 2
02_Hum3	2002	Human	Faeces	Denmark	Denmark	32	Sensitive			cluster 2
02_Hum4	2002	Human	Faeces	Greece	Denmark	32	Sensitive			cluster 2
02_Swine1	2002	Swine	Fresh meat	Denmark	Denmark	32	Sensitive			cluster 2
02_Swine2	2002	Swine	Fresh meat	Denmark	Denmark	32	A	blaTEM-1B	IncI1	cluster 2
03_Hum1	2003	Human	Culture	Denmark	Denmark	32	Sensitive			cluster 3
03_Hum2	2003	Human	Culture	Unknown	Denmark	1823	Sensitive			Distant ST
04_Environ	2004	Environment	Water	Benin	Benin	603	Sensitive			Distant ST
04_Food	2004	Food	Food	Germany	Germany	1032	Sensitive			cluster 5
04_Hum1	2004	Human	Faeces	Denmark	Denmark	32	Sensitive			cluster 2
04_Hum2	2004	Human	Faeces	Denmark	Denmark	32	Sensitive			cluster 2
04_Hum3	2004	Human	Faeces	Denmark	Denmark	32	Sensitive			cluster 2
04_Hum4	2004	Human	Faeces	Denmark	Denmark	32	Sensitive			cluster 1
04_Hum5	2004	Human	Faeces	Caribbean	Denmark	32	Sensitive			cluster 2
04_Hum6	2004	Human		Taiwan	Taiwan	603	Sensitive			Distant ST
04_Swine	2004	Swine		Ireland	Ireland	32	ASSuTTm	aph(3'')-Ib,aph(6)-Id,aadA1,blaTEM-1B,mph(B),sul1,sul2,tet(B),dfrA1	IncHI2A,IncHI2,TrfA,IncQ1	cluster 3
05_Avian1	2005	Avian		Denmark	Denmark	32	Sensitive			cluster 1
05_Avian2	2005	Avian	Fresh meat	Denmark	Denmark	32	Sensitive			cluster 8
05_Feed	2005	Feed	Feed	Denmark	Denmark	32	Sensitive			cluster 3
05_Swine1	2005	Swine	Intestine	Denmark	Denmark	1824	Sensitive			cluster 7
05_Swine2	2005	Swine		Denmark	Denmark	32	Sensitive			cluster 1
06_Avian	2006	Avian	Imported chicken	Denmark*	Denmark*	32	SSuTCiNx	aadA1,sul1,tet(A),gyrA p.S83Y,parC p.T57S	IncFIB(pENTAS01)-pSEI-like	cluster 1
06_Bovine	2006	Bovine	Liver	Denmark	Denmark	32	Sensitive			cluster 2
06_Environ	2006	Environment	River	USA	USA	32	Sensitive			cluster 3
06_Feed1	2006	Feed	Feed	Denmark	Denmark	32	Sensitive			cluster 2
06_Feed2	2006	Feed	Animal feed	United Kingdom	Northern Ireland	32	Sensitive			cluster 8
06_Hum1	2006	Human		Netherlands	Netherlands	32-hisD deleted	Sensitive		Inc1	cluster 2
06_Hum2	2006	Human	Blood	Ireland	Ireland	32	SSuTCiNx	aadA1,sul1,tet(A),gyrA p.S83Y,parC p.T57S	IncFIB(pENTAS01)-pSEI-like	cluster 1
06_Hum3	2006	Human	Faeces	India	Denmark	1825	ATTmCiNx	tet(A),blaTEM-1B,dfrA1,gyrA p.S83F,parC p.T57S		cluster 3
06_Swine	2006	Swine	pork	Denmark	Denmark	32	Sensitive		IncX1, Col156	cluster 4
07_Avian	2007	Avian	Chicken	United Kingdom	Northern Ireland	32	Sensitive			cluster 8
07_Feed	2007	Feed	Feed	Denmark	Denmark	32	Sensitive			cluster 7
07_Hum1	2007	Human		Taiwan	Taiwan	32	Sensitive			cluster 2
07_Hum2	2007	Human	Faeces	Iran	Denmark	32	SSuTTmNeCiNx	aadA1,aph(3')-Ia,sul1,tet(A),dfrA14,gyrA p.D87N,parC p.T57S	IncFIB(pENTAS01)-pSEI-like	cluster 1
07_Hum3	2007	Human	Faeces	Unknown	Denmark	32	SSuTTmNeCiNx	aadA1,aph(3')-Ia,sul1,tet(A),dfrA14,gyrA p.S83Y,parC p.T57S	IncFIB(pENTAS01)-pSEI-like	cluster 1
07_Hum4	2007	Human	Faeces	Denmark	Denmark	32	CiNx	gyrA p.S83F,parC p.T57S		cluster 2
07_Hum5	2007	Human	Faeces	Morocco	Denmark	32	Sensitive			cluster 2
07_Swine1	2007	Swine	Lungs	Denmark	Denmark	32	Sensitive			cluster 7
07_Swine2	2007	Swine	lymphnode	Denmark	Denmark	32	Sensitive			cluster 7
08_Avian	2008	Avian	Imported duck	Denmark*	Denmark*	32	Sensitive			cluster 1
08_Environ1	2008	Environment	Stable environment-poultry	Denmark	Denmark	32	Sensitive			cluster 2
08_Environ2	2008	Environment	Stable environment-poultry	Denmark	Denmark	32	Sensitive		Col8282	cluster 2
08_Hum1	2008	Human	Faeces	Venezuela	Denmark	32	Sensitive			cluster 1
08_Hum2	2008	Human	Faeces	Turkey	Denmark	32	SSuTTmNeCiNx	aadA1,aph(3')-Ia,sul1,tet(A),dfrA14,gyrA p.S83Y,parC p.T57S	IncFIB(pENTAS01)-pSEI-like	cluster 1
08_Hum3	2008	Human	Faeces	Cuba	Denmark	32	Sensitive			cluster 2
08_Hum4	2008	Human	Faeces	South Africa	Denmark	32	CSSuTFfn	aph(3'')-Ib,aph(6)-Id,floR,sul2,tet(A)	IncA/C2	cluster 8
08_Hum5	2008	Human	Faeces	Unknown	Denmark	32	CiNx	gyrA p.S83F,parC p.T57S		cluster 2
08_Hum6	2008	Human	Faeces	Bulgaria	Denmark	32	Sensitive			cluster 3
08_Hum7	2008	Human	Faeces	Tunisia	Denmark	32	Sensitive			cluster 3
08_Hum8	2008	Human	Faeces	Greece	Denmark	32	SSuTCiNx	aadA1,sul1,tet(A),gyrA p.S83Y,parC p.T57S	IncFIB(pENTAS01)-pSEI-like	cluster 1
08_Swine1	2008	Swine	Imported pork	Denmark*	Denmark*	32	Sensitive		IncFIB(pHCM2)	cluster 7
08_Swine2	2008	Swine		Denmark	Denmark	32	ASSuTm	aadA1,aph(3'')-Ib,aph(6)-Id,blaTEM-1B,sul1,sul2,dfrA1	IncI1,IncQ1	cluster 4
09_Bovine1	2009	Bovine	Raw Meat	Ireland	Ireland	32	ASuTmCiNx	blaTEM-1B,sul2,dfrA1,gyrA p.S83Y,parC p.T57S	IncI1	cluster 3
09_Bovine2	2009	Bovine	Fresh meat	Denmark*	Denmark*	32	Sensitive			cluster 7
09_Environ1	2009	Environment	Stable environment-poultry	Denmark	Denmark	32	Sensitive			cluster 4
09_Environ2	2009	Environment	River (Thames River)	United Kingdom	United Kingdom	32	Sensitive			cluster 6
09_Hum1	2009	Human	Faeces	Pakistan	Denmark	32	ACSuTTmGCiNx	ant(2'')-Ia,blaTEM-1B,cmlA1,sul1,tet(A),dfrA1,gyrA p.D87Y,parC p.T57S		cluster 3
09_Hum2	2009	Human	Faeces	Thailand	Denmark	32	Sensitive			cluster 7
09_Hum3	2009	Human	Faeces	Egypt	Denmark	32	SSuTCiNx	aadA1,sul1,tet(A),gyrA p.D87G,parC p.T57S	IncFIB(pENTAS01)-pSEI-like	cluster 1
09_Hum4	2009	Human	Culture	Denmark	Denmark	32	Sensitive			cluster 6
09_Hum5	2009	Human	Faeces	Denmark	Denmark	32	CiNx	gyrA p.S83F,parC p.T57S		cluster 2
09_Plant	2009	Plant	Soya Spruce	Netherlands	Netherlands	32	Sensitive			cluster 3
09_Swine	2009	Swine	Fresh meat	Denmark	Denmark	32	Sensitive			cluster 4
10_Avian	2010	Avian		Denmark	Denmark	32	Sensitive			cluster 2
10_Environ	2010	Environment	Stable environment-poultry	Denmark	Denmark	32	Sensitive			cluster 8
10_Feed	2010	Feed	Feed	Denmark	Denmark	32	Sensitive			cluster 1
10_Hum1	2010	Human	Faeces	Denmark	Denmark	32	Sensitive			cluster 1
10_Hum10	2010	Human	Faeces	Egypt	Denmark	32	Sensitive			cluster 1
10_Hum2	2010	Human	Faeces	Denmark	Denmark	32	Sensitive			cluster 1
10_Hum3	2010	Human	Faeces	Denmark	Denmark	32	Sensitive			cluster 1
10_Hum4	2010	Human	Faeces	Denmark	Denmark	32	S			cluster 1
10_Hum5	2010	Human	Faeces	Denmark	Denmark	32	Sensitive			cluster 1
10_Hum6	2010	Human	Faeces	Lebanon	Denmark	32	Sensitive			cluster 2
10_Hum7	2010	Human	Faeces	Poland	Denmark	32	SSuTCiNx	aadA1,sul1,tet(A),gyrA p.S83Y,parC p.T57S	IncFIB(pENTAS01)-pSEI-like	cluster 1
10_Hum8	2010	Human	Faeces	Iran	Denmark	32	SSuTmCiNx	aadA1,sul1,dfrA14,gyrA p.D87N,parC p.T57S	IncFIB(pENTAS01)-pSEI-like	cluster 1
10_Hum9	2010	Human	Faeces	Denmark	Denmark	32	SSuTmG	aadA1,aac(3)-VIa,dfrA1,sul1	IncI1	cluster 1
10_Swine	2010	Swine	Fresh meat	Denmark	Denmark	32	Sensitive			cluster 8
11_Avian	2011	Avian	Fresh meat	Denmark	Denmark	32	SSuTCiNx	aadA1,sul1,tet(A),gyrA p.S83Y,parC p.T57S	IncFIB(pENTAS01)-pSEI-like	cluster 1
11_Feed	2011	Feed	Feed	Denmark	Denmark	32	Sensitive			cluster 3
11_Hum1	2011	Human	Faeces	Nepal	Denmark	32	SSuTTmNeCiNx	aadA1,aph(3')-Ia,sul1,tet(A),dfrA14,gyrA p.D87G,parC p.T57S	IncFIB(pENTAS01)-pSEI-like	cluster 1
11_Hum2	2011	Human	Faeces	Turkey	Denmark	32	SSuTTmCiNx	aadA1,sul1,tet(A),dfrA14,gyrA p.S83Y,parC p.T57S	IncFIB(pENTAS01)-pSEI-like	cluster 1
11_Hum3	2011	Human	Faeces	India	Denmark	32	SSuTTmNeCiNx	aadA1,aph(3')-Ia,sul1,tet(A),dfrA14,gyrA p.D87G,parC p.T57S	IncFIB(pENTAS01)-pSEI-like	cluster 1
11_Hum4	2011	Human	Faeces	India	Denmark	32	CSuGCiNx	ant(2'')-Ia,cmlA1,sul1,gyrA p.D87Y,parC p.T57S	IncI1	cluster 3
11_Hum5	2011	Human	Faeces	Denmark	Denmark	32	Sensitive			cluster 4
11_Hum6	2011	Human	Faeces	Unknown	Denmark	32	SSuTCiNx	aadA1,sul1,tet(A),gyrA p.S83Y,parC p.T57S	IncFIB(pENTAS01)-pSEI-like	cluster 1
11_Hum7	2011	Human	Faeces	South Africa	Denmark	603	Sensitive			Distant ST
12_Environ	2012	Environment	Stable environment-poultry	Denmark	Denmark	32	ASTNe	aph(3')-Ia, aph(3'')-Ib,aph(6)-Id,blaTEM-1A,tet(B)	IncI1,IncX4	cluster 1
12_Hum1	2012	Human	Faeces	Denmark	Denmark	32	Sensitive			cluster 3
12_Hum2	2012	Human	Faeces	Denmark	Denmark	32	Sensitive			cluster 3
85_Hum	1985	Human	Human carrier	Germany	Germany	32	Sensitive			cluster 3
98_Reptile	1998	Reptile	Snake	Germany	Germany	32	Sensitive			cluster 2
99_Avian	1999	Avian	Chicken meat	Japan	Japan	32	SSuTTmNe	aadA1,aph(3')-Ia,sul1,tet(A),dfrA14	IncFIB(pENTAS01)-pSEI-like	cluster 1
99_Environ	1999	Environment	GP center (egg packaging)	Japan	Japan	32	Sensitive			cluster 2
99_Hum	1999	Human	Faeces	Greece	Denmark	32	Sensitive			cluster 3
Kauffman1	Unknown	Unknown		Unknown	Unknown	32	Sensitive			cluster 2
Kauffman3	1961	Unknown		Unknown	Unknown	32	Sensitive			cluster 3
Kauffman4	1943	Unknown	Unknown	Unknown	Unknown	493	Sensitive			Distant ST
SARB26	Unknown	Human	Human	USA	USA	32	Sensitive			cluster 2

*** - imported meat**

**Resistance abriviations**
A = Ampillicin
C = Chloramphenicol
Ci = Ciprofloxacin
Ffn = Florfenicol
G = Gentamicin
Nx = Nalidixic acid
Ne = Neomycin
S = Streptomycin
Su = Sulfamethoxazole
T = Tetracycline
Tm = Trimethoprim

Table S2

Q-values for Structure analysis

* Strain with questionable Q-values

Main lineage						
Strain ID	Cluster	Fst_1	Fst_2	Fst_3	Fst_4	Fst_5
04_Hum4*	cluster 1	0.010	0.001	0.692	0.262	0.034
10_Feed*	cluster 1	0.031	0.000	0.679	0.272	0.017
12_Environ*	cluster 1	0.007	0.001	0.690	0.289	0.013
08_Hum1*	cluster 1	0.079	0.001	0.685	0.226	0.009
05_Swine2*	cluster 1	0.020	0.000	0.675	0.292	0.012
10_Hum1	cluster 1	0.000	0.000	0.996	0.002	0.001
10_Hum2	cluster 1	0.002	0.000	0.994	0.002	0.002
10_Hum3	cluster 1	0.000	0.000	0.997	0.001	0.001
10_Hum4	cluster 1	0.000	0.000	0.997	0.001	0.001
10_Hum5	cluster 1	0.001	0.000	0.995	0.002	0.001
00_Poultry	cluster 1	0.081	0.000	0.915	0.001	0.002
06_Poultry	cluster 1	0.083	0.000	0.915	0.000	0.001
08_Avian	cluster 1	0.079	0.000	0.917	0.003	0.001
05_Poultry1	cluster 1	0.011	0.000	0.987	0.001	0.001
99_Poultry	cluster 1	0.073	0.000	0.923	0.002	0.001
06_Hum2	cluster 1	0.044	0.000	0.955	0.000	0.001
11_Poultry	cluster 1	0.124	0.000	0.874	0.001	0.001
07_Hum2	cluster 1	0.077	0.000	0.919	0.002	0.001
07_Hum3	cluster 1	0.146	0.000	0.850	0.002	0.001
08_Hum2	cluster 1	0.111	0.000	0.885	0.002	0.001
09_Hum3	cluster 1	0.126	0.000	0.870	0.001	0.003
10_Hum7	cluster 1	0.078	0.000	0.920	0.001	0.000
10_Hum8	cluster 1	0.161	0.000	0.836	0.002	0.001
10_Hum9	cluster 1	0.000	0.000	0.997	0.002	0.001
10_Hum10	cluster 1	0.001	0.000	0.996	0.002	0.001
11_Hum1	cluster 1	0.126	0.000	0.871	0.002	0.001
11_Hum2	cluster 1	0.128	0.000	0.868	0.002	0.001
11_Hum3	cluster 1	0.118	0.000	0.878	0.002	0.001
11_Hum6	cluster 1	0.130	0.000	0.868	0.001	0.001
08_Hum8	cluster 1	0.074	0.000	0.925	0.001	0.001
02_Hum2	cluster 2	0.000	0.000	0.001	0.998	0.001
02_Hum3	cluster 2	0.000	0.000	0.001	0.997	0.001
04_Hum1	cluster 2	0.007	0.000	0.002	0.990	0.001
04_Hum2	cluster 2	0.004	0.000	0.002	0.992	0.002
04_Hum3	cluster 2	0.060	0.000	0.001	0.938	0.001
06_Feed1	cluster 2	0.005	0.000	0.002	0.991	0.001
08_Environ1	cluster 2	0.083	0.000	0.006	0.905	0.005
10_Poultry	cluster 2	0.044	0.001	0.003	0.951	0.002
02_Swine1	cluster 2	0.000	0.000	0.001	0.997	0.001
99_Environ	cluster 2	0.116	0.000	0.007	0.874	0.003
06_Hum1	cluster 2	0.019	0.000	0.003	0.976	0.001
SARB26	cluster 2	0.044	0.001	0.004	0.951	0.001
07_Hum1	cluster 2	0.055	0.000	0.004	0.937	0.003
98_Reptile	cluster 2	0.001	0.000	0.003	0.994	0.003
00_Reptile	cluster 2	0.000	0.000	0.001	0.997	0.002
02_Swine2	cluster 2	0.000	0.000	0.001	0.997	0.001
06_Bovine	cluster 2	0.033	0.000	0.003	0.962	0.002
Kauffman1	cluster 2	0.061	0.000	0.003	0.933	0.002
08_Environ2	cluster 2	0.082	0.001	0.006	0.901	0.010
04_Hum5	cluster 2	0.103	0.000	0.012	0.881	0.004
02_Hum4	cluster 2	0.002	0.000	0.001	0.996	0.001
07_Hum4	cluster 2	0.001	0.000	0.001	0.998	0.001
08_Hum3	cluster 2	0.053	0.000	0.007	0.938	0.001
08_Hum5	cluster 2	0.000	0.000	0.001	0.997	0.001
10_Hum6	cluster 2	0.054	0.000	0.004	0.940	0.002
07_Hum5	cluster 2	0.002	0.001	0.001	0.994	0.002
09_Hum5	cluster 2	0.000	0.000	0.001	0.997	0.001
02_Hum1	cluster 3	0.034	0.000	0.001	0.003	0.962
12_Hum1	cluster 3	0.000	0.000	0.001	0.001	0.998
12_Hum2	cluster 3	0.000	0.000	0.001	0.001	0.998
09_Plant	cluster 3	0.000	0.000	0.001	0.001	0.997
09_Bovine1	cluster 3	0.077	0.000	0.004	0.005	0.915
04_Swine	cluster 3	0.071	0.000	0.001	0.005	0.923
06_Environ	cluster 3	0.115	0.001	0.003	0.006	0.875
85_Hum*	cluster 3	0.124	0.001	0.006	0.426	0.444
05_Feed	cluster 3	0.046	0.000	0.001	0.002	0.950
06_Hum3	cluster 3	0.019	0.000	0.001	0.001	0.978
00_Hum1	cluster 3	0.082	0.000	0.003	0.004	0.911
Kauffman3	cluster 3	0.030	0.001	0.005	0.006	0.959
03_Hum1	cluster 3	0.082	0.000	0.002	0.002	0.913
08_Hum6	cluster 3	0.059	0.000	0.002	0.002	0.936
08_Hum7	cluster 3	0.072	0.000	0.001	0.003	0.923
09_Hum1	cluster 3	0.006	0.000	0.001	0.001	0.992
11_Hum4	cluster 3	0.043	0.000	0.001	0.001	0.954
99_Hum	cluster 3	0.031	0.000	0.002	0.001	0.965
11_Feed	cluster 3	0.005	0.000	0.001	0.001	0.993
00_Hum2	cluster 3	0.034	0.001	0.001	0.002	0.962
06_Swine	cluster 4	0.089	0.852	0.013	0.016	0.029
08_Swine	cluster 4	0.001	0.998	0.000	0.000	0.001
09_Environ1	cluster 4	0.001	0.998	0.000	0.000	0.001
11_Hum5	cluster 4	0.068	0.928	0.001	0.002	0.001
09_Swine	cluster 4	0.003	0.995	0.000	0.001	0.000
04_Food*	cluster 5	0.203	0.465	0.002	0.186	0.144
09_Environ2*	cluster 6	0.001	0.687	0.004	0.228	0.080
09_Hum4*	cluster 6	0.001	0.687	0.006	0.196	0.109

Distant lineage			
Strain ID	Cluster	Fst_1	Fst_2
08_Food	cluster 7	0.000	1.000
05_Swine1	cluster 7	0.001	0.999
07_Swine1	cluster 7	0.001	0.999
07_Swine2	cluster 7	0.001	0.999
07_Feed	cluster 7	0.000	1.000
09_Bovine2	cluster 7	0.000	1.000
09_Hum2	cluster 7	0.001	0.999
10_Environ	cluster 8	1.000	0.000
06_Feed2	cluster 8	1.000	0.000
07_Poultry	cluster 8	1.000	0.000
02_Poultry	cluster 8	1.000	0.000
05_Poultry2	cluster 8	1.000	0.000
10_Swine	cluster 8	1.000	0.000
08_Hum4	cluster 8	0.999	0.001
00_Hum3	cluster 8	0.999	0.001
