# Supplementary figures and images for: WGS based study of the population structure of Salmonella enterica serovar Infantis
Source: BMC Genomics. 2019 Nov 15;20:870. doi: 10.1186/s12864-019-6260-6 (PMC6858691; doi:10.1186/s12864-019-6260-6)

Figure S1

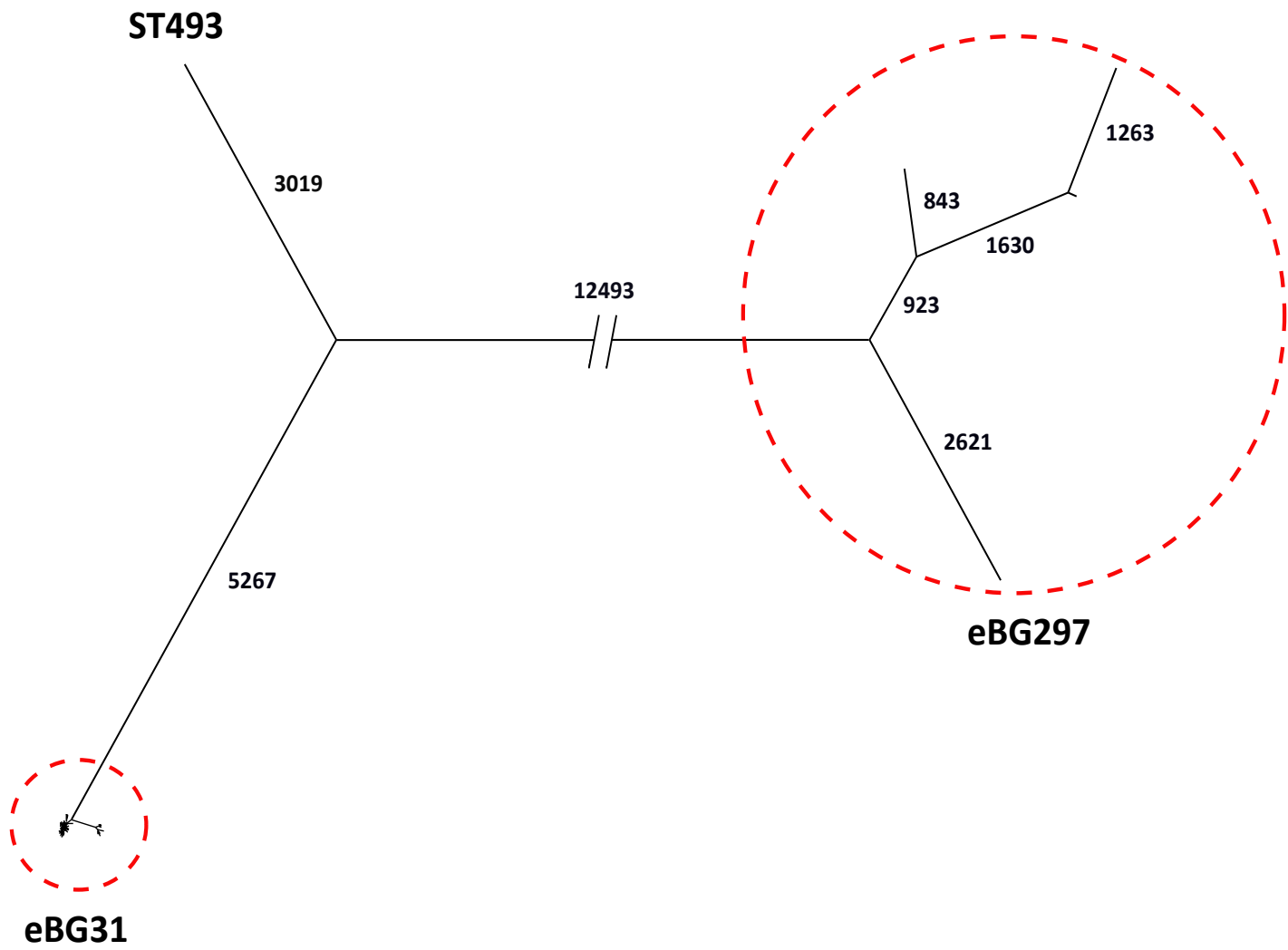

Figure S2

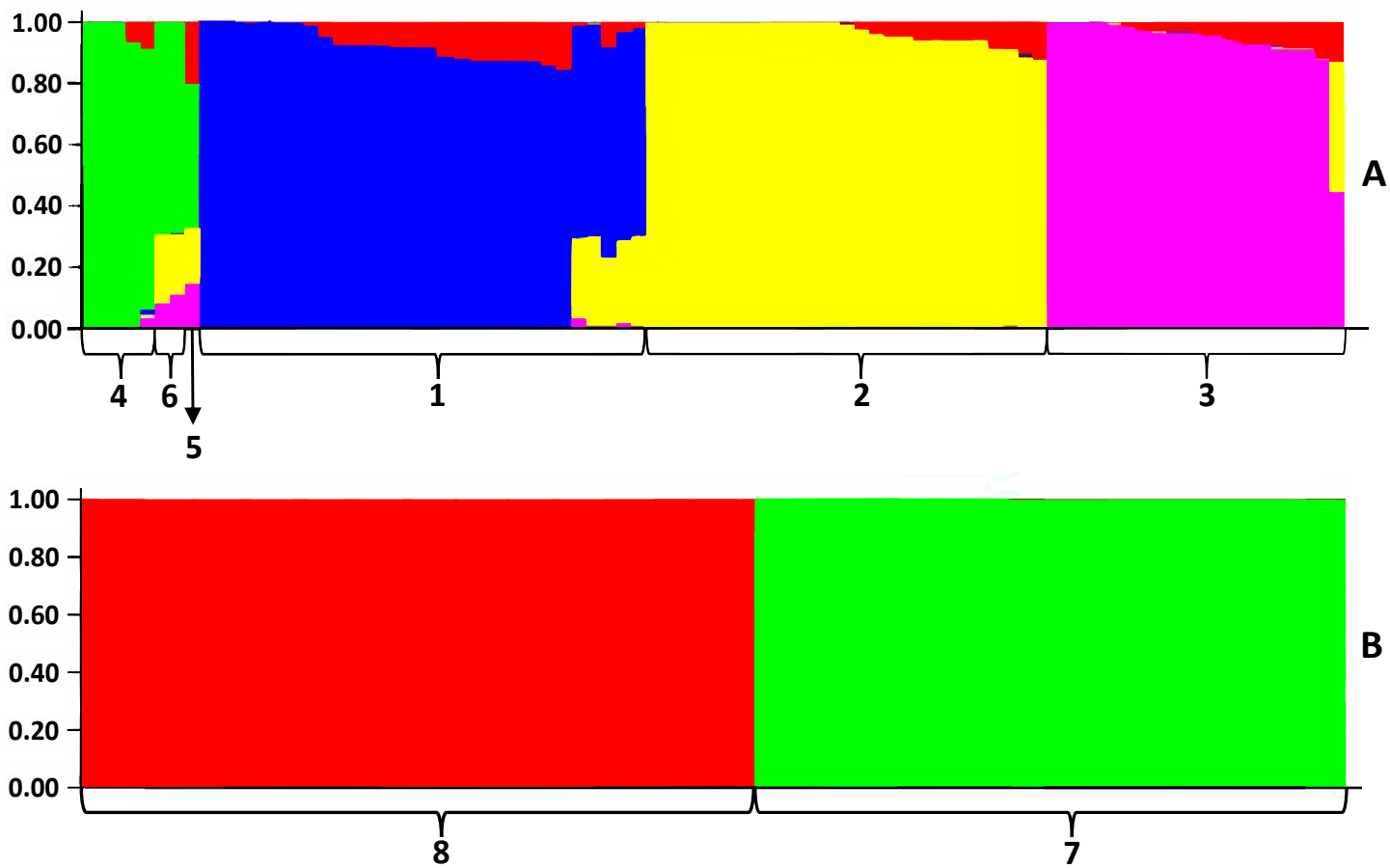

Figure S3

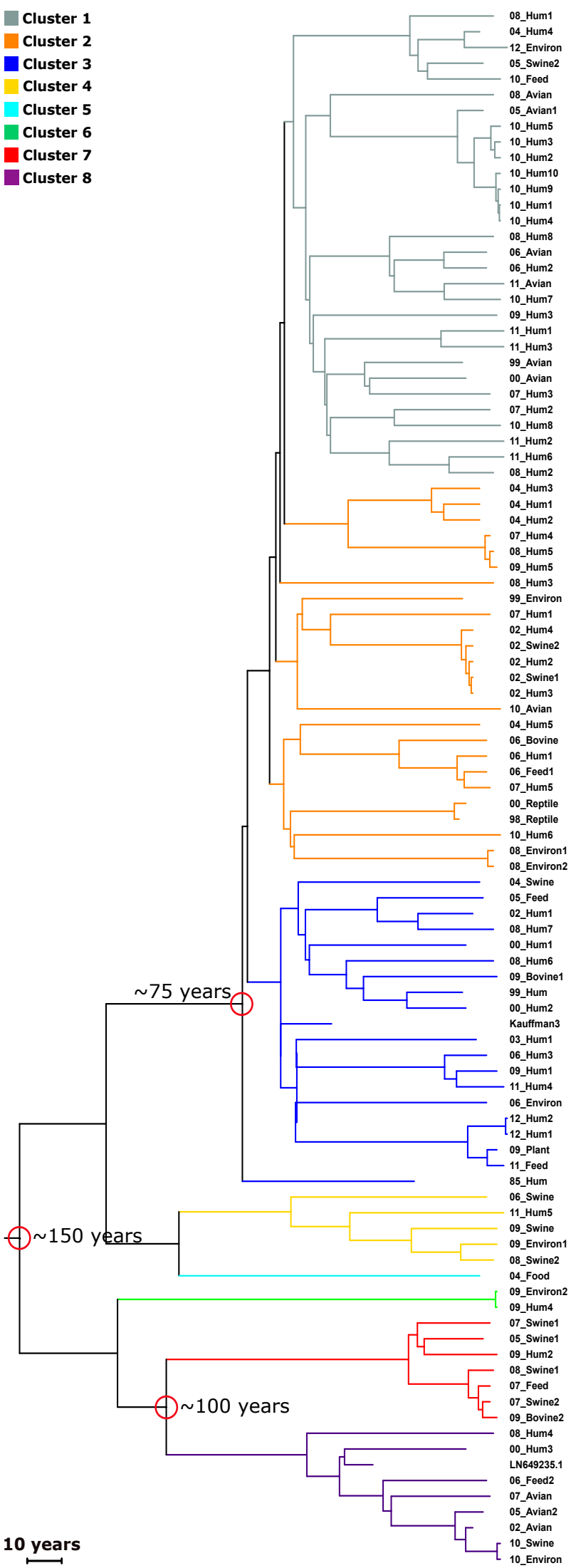

Figure S4

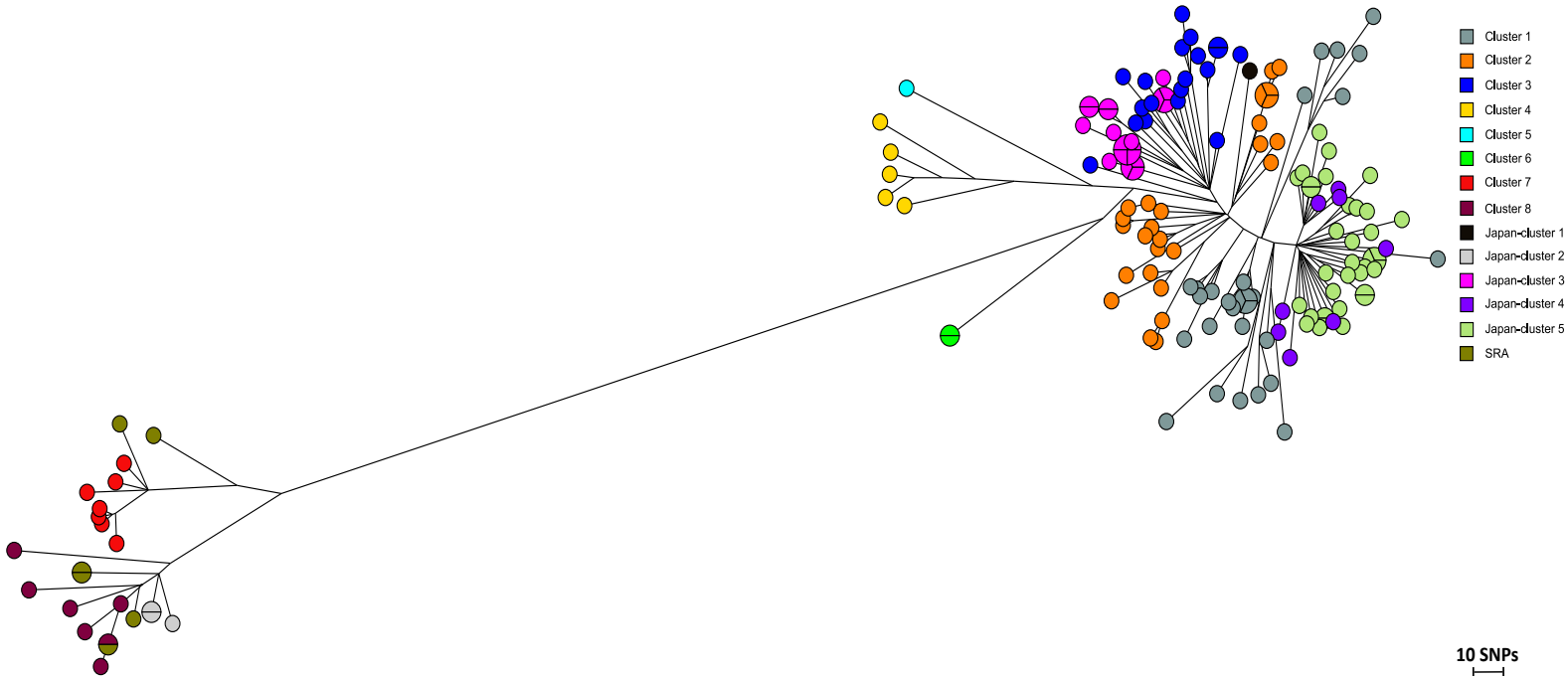

Figure S5

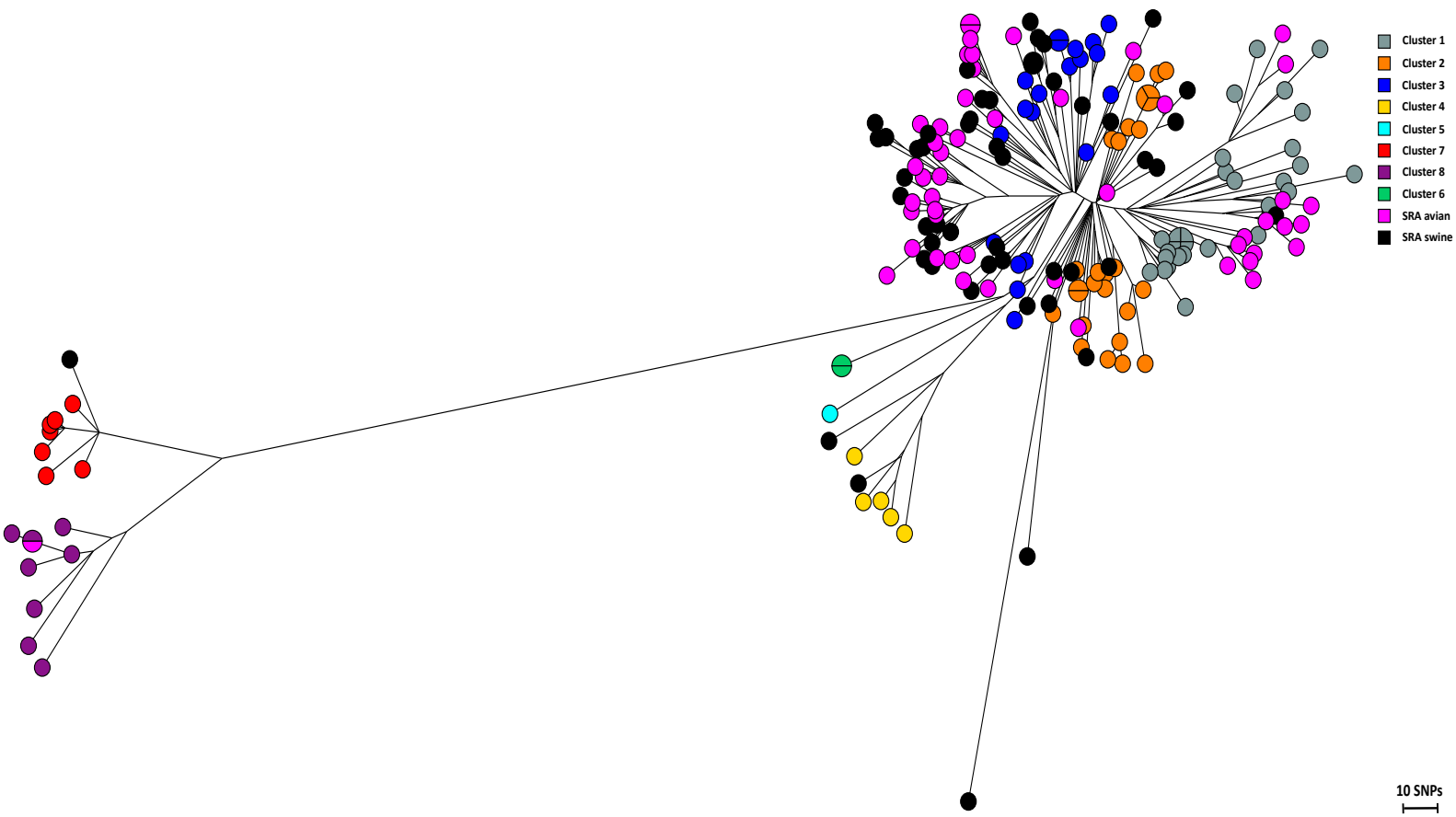

Figure S6

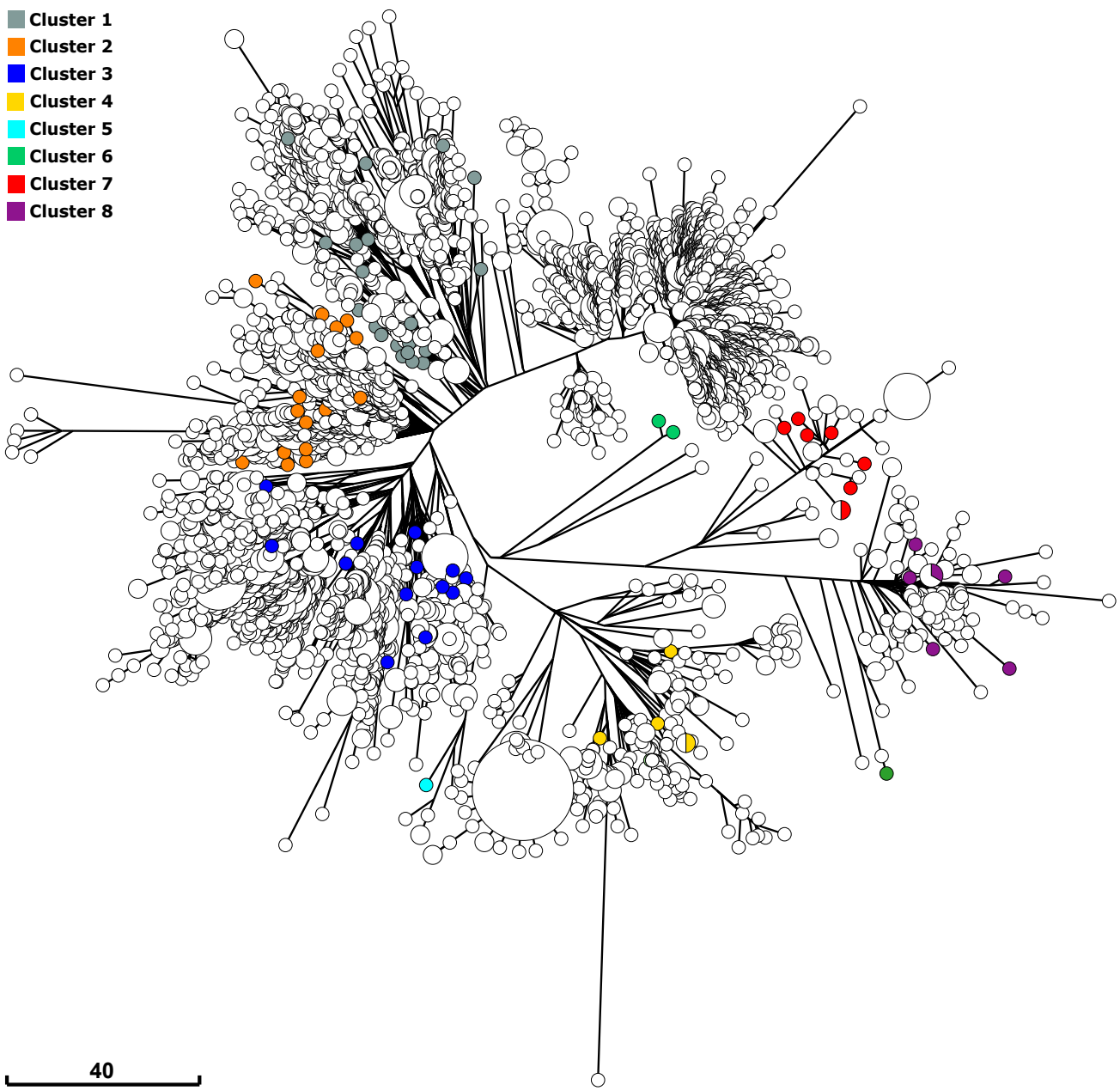

Supplement: Supplementary file 2 — Additional file 2: Figure S1. Maximum parsimony tree of 105 strains of Salmonella Infantis based on 28,860 core-genome SNPs with Salmonella Infantis CVM44454 as the reference genome. Branches are labelled with the number of SNP differences. Strains belonging to E-Burst Group (eBG) 31 and 297 are marked in red circles. Figure S2. Q-plots based on probability values (Q) from STRUCTURE analysis of 2311 core-genome SNPs identified in 100 Salmonella Infantis strains with Salmonella Infantis CVM44454 as the reference genome. Genetic clusters are marked with curly brackets and cluster number. A: STRUCTURE analysis on main lineage with 85 strains B: STRUCTURE analysis on distant lineage with 15 strains. Figure S3. Mean evolutionary tree calculated from BEAST analysis with the best-fitted substitution model (GTR-BS-R) on 2311 core-genome SNPs. Branches are coloured according to clusters and branch length correlates with time in years. Figure S4. Maximum parsimony tree of 167 strains of Salmonella Infantis based on 3454 core-genome SNPs with Salmonella Infantis CVM44454 as the reference genome. The collection of strains includes the 100 strains examined in this study, all genomes from Yokoyama et al. [23] (labelled Japan-clusters) and additional 6 genomes from SRA belonging to the distant lineage (cluster 7 and 8). Nodes are coloured according to clusters. Figure S5. Maximum parsimony tree of 200 genomes of Salmonella Infantis based on 4079 core-genome SNPs with Salmonella Infantis CVM44454 as the reference genome. The collection includes the 100 strains examined in this study and additional 50 genomes isolated from avian sources and 50 genomes from swine (downloaded from SRA). Nodes are coloured according to clusters and source. Figure S6. Core genome-derived phylogeny of 105 strains of Salmonella Infantis including additional 7852 public available S. Infantis genomes from Enterobase. RapidNJ tree based on cgMLST. Nodes are coloured according to clusters defined in this stud [file 12864_2019_6260_MOESM2_ESM.pdf]
